# Serum Pregenomic RNA Combined With Hepatitis B Core-Related Antigen Helps Predict the Risk of Virological Relapse After Discontinuation of Nucleos(t)ide Analogs in Patients With Chronic Hepatitis B

**DOI:** 10.3389/fmicb.2022.901233

**Published:** 2022-06-22

**Authors:** Fa-Da Wang, Jing Zhou, Lan-Qing Li, Meng-Lan Wang, Ya-Cao Tao, Yong-Hong Wang, Dong-Mei Zhang, En-Qiang Chen

**Affiliations:** ^1^Center of Infectious Diseases, West China Hospital, Sichuan University, Chengdu, China; ^2^Department of Infectious Disease, Chengdu Third People’s Hospital, Chengdu, China

**Keywords:** chronic hepatitis B, discontinuation, hepatitis B core-associated antigen, nucleos(t)ide analogs, pregenomic RNA

## Abstract

**Background and Aim:**

Cessation of nucleos(t)ide analogs (NAs) therapy in patients with chronic hepatitis B (CHB) is uncommon. Although criteria for discontinuation appear in some guidelines, the indicators for assessing discontinuation of NAs are limited, whether NAs can be safely ceased remains a difficult clinical issue. Our study aimed to investigate the role of serum pregenomic RNA (pgRNA) and hepatitis B core-related antigen (HBcrAg) at the end of treatment (EOT) in guiding the safe discontinuation of NAs in CHB patients.

**Methods:**

This is a retrospective study, clinical data of all CHB patients who discontinued NAs treatment at West China Hospital between June 2020 and January 2021 were collected, including EOT pgRNA, HBcrAg, hepatitis B surface antigen (HBsAg), etc. All patients should meet the Asian-Pacific guideline for discontinuation. Observing virological relapse (VR) rates during 1 year of NAs discontinuation and analyzing the relationship between EOT pgRNA, HBcrAg, and VR.

**Results:**

A total of 64 patients were enrolled in this study and 33 (51.5%) patients experienced VR in 1 year. EOT pgRNA positivity (OR = 14.59, *p* = 0.026) and EOT higher HBcrAg levels (OR = 14.14, *p* = 0.001) were independent risk factors for VR. The area under the receiver-operating characteristic (AUROC) value of EOT HBcrAg for VR was 0.817 (*p* < 0.001), optimal cut-off value was 3.3 log10 U/mL. Patients with EOT pgRNA positivity and EOT HBcrAg >3.3 log10 U/mL were more likely to experience VR after discontinuation of NAs (88.9 vs. 45.5%, *p* = 0.027).

**Conclusion:**

According to current guidelines, a higher VR rate occurs after cessation of NAs. EOT pgRNA positivity and higher HBcrAg level carries a higher risk of VR. Combining these novel markers can better help us assess whether patients can safely cease NAs treatment.

## Introduction

HBV-infected patients are at increased risk of cirrhosis, liver failure and hepatocellular carcinoma (HCC), nearly one million people die each year from HBV-related end-stage liver disease and its complications (Cooke et al., 2019). Due to the presence of intrahepatocellular covalently closed circular DNA (cccDNA), a long course of antiviral therapy is required for chronic hepatitis B (CHB) patients. Long-term nucleos(t)ide analogs (NAs) therapy can improve liver histology and reduce the risk of liver-related complications through sustained viral suppression. However, the financial burden, patient compliance, and drug side effects have made the question of whether NAs therapy can be discontinued in CHB patients become a hot clinical issue.

Although criteria for discontinuation of NAs appear in some guidelines, the indicators for assessing discontinuation are limited. The ideal endpoint for NAs therapy in patients with CHB is the loss of hepatitis B surface antigen (HBsAg), but it can only be achieved in a very small number of patients and requires decades of treatment (Chevaliez et al., 2013). To address this issue, the Liver Association guidelines suggest alternative endpoints to NAs therapy (Liaw et al., 2012; Terrault et al., 2016). The Asian-Pacific guideline recommend that in hepatitis B e antigen (HBeAg) positive patients, NAs treatment can be stopped when HBeAg seroconversion with undetectable HBV DNA has been maintained for at least 12 months. In HBeAg-negative patients, the treatment discontinuation can be considered if patients have been treated for at least 2 years with undetectable HBV DNA documented on three separate occasions 6 months. Following Asian-Pacific guideline, studies have demonstrated that very high rates of virological relapse (VR) after discontinuation and recommended continued NAs treatment (Chaung et al., 2012; Chi et al., 2015; Seto et al., 2015). Hence, we need comprehensive indicators to guide safe discontinuation of NAs treatment for CHB patients.

Hepatitis B core-related antigen (HBcrAg) and pregenomic RNA (pgRNA) are two novel serological indicators for patients with CHB. Studies have shown that HBcrAg is a surrogate marker of both intrahepatic cccDNA and its transcriptional activity (Testoni et al., 2019). pgRNA is a direct transcription product of HBV cccDNA, and is further reverse transcribed by the reverse transcriptase activity of the polymerase into a new relaxed circular DNA (rcDNA) genome, eventually leading to replenishment of the cccDNA pool and formation of HBV DNA-containing virions (Lin et al., 2020). NAs act mainly on reverse transcriptase and do not interfere with pgRNA synthesis, so pgRNA is a good indicator of cccDNA activity in the liver (Trépo et al., 2014). The new serum biomarkers pgRNA and hepatitis B core-associated antigen (HBcrAg) have a role in quantifying cccDNA (Ghany et al., 2021), so they can be used to evaluate the risk of viral reactivation after discontinuation of NAs.

In this study, we established a retrospective cohort to examine VR rates up to 1 year after discontinuation of NAs in CHB patients and to explore the factors associated with successful discontinuation of NAs treatment, particularly end of treatment (EOT) HBsAg, pgRNA, and HBcrAg levels.

## Materials and Methods

### Patients

**FIGURE 1:**
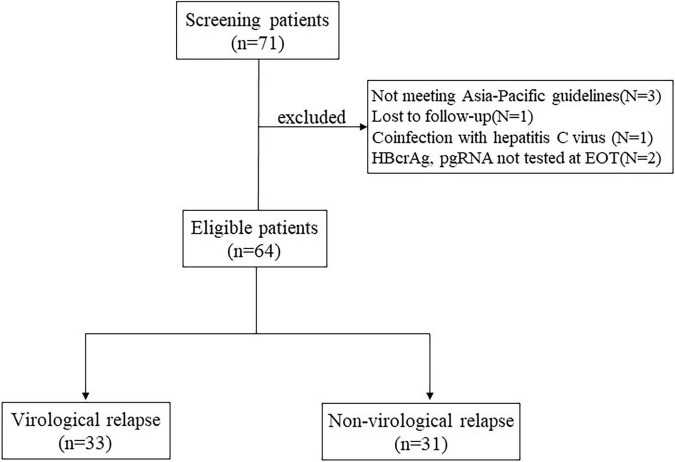
The patients screening and enrollment flow chart.

This is a retrospective study, the study subjects were all CHB patients who discontinued NAs treatment at West China Hospital of Sichuan University between June 2020 and January 2021, including those who were lost to follow-up. HBeAg-positive and HBeAg-negative patients who meet the Asia-Pacific guidelines of NAs discontinuation criteria are eligible for inclusion. HBeAg positive patients were required to achieve HBeAg seroconversion and undetectable HBV DNA for at least 12 months of consolidation therapy. HBeAg negative patients were required to achieve undetectable HBV DNA and then at least 18 months of intensive treatment (Liaw et al., 2012; Terrault et al., 2016). Additional eligibility criteria were age ≥18 years, HBsAg positivity and undetectable HBV DNA at the time of NAs discontinuation. Exclusion criteria included coinfection with hepatitis C virus, hepatitis D virus, or human immunodeficiency virus, immunocompromised status or malignancy, autoimmune liver disease, alcohol abuse, previous history of liver transplantation, and other severe or active disease. History of decompensated liver disease or presence of decompensated cirrhosis (Figure 1).

This study was conducted in accordance with the 1975 Declaration of Helsinki. The study protocol was approved by the West China Hospital Ethics Committee, and informed consent was obtained from each patient.

### Follow-Up, Endpoint

When NAs treatment was discontinued, each patient was tested for pgRNA, HBcrAg and other indicators. Patients were followed up every 3 months after discontinuation for a minimum of 12 months or VR, with biochemical and virological tests performed at each follow-up visit. The primary study endpoint was VR within 1 year of discontinuation, defined as HBV DNA levels >2,000 IU/mL with or without elevated alanine aminotransferase (ALT). Patients with VR had been restarted on NAs therapy and were managed according to CHB guidelines.

### Laboratory Testing

Serum biochemical indexes were measured according to standard procedures (Olympus AU5400, Olympus Corporation, Tokyo, Japan). Serum HBsAg levels were quantitatively measured using an Elecsys^®^ HBsAg II Quant Assay (Roche Diagnostics, Penzberg, Germany). Serum HBeAg status was assessed using electrochemiluminescence immunoassay (Roche Diagnostics, Indianapolis, IN, United States). Serum HBV DNA concentration was quantitatively determined using a Cobas TaqMan assay kit (Roche Diagnostics, Branchburg, NJ, United States), with a lower limit of detection of 20 IU/mL.

### Serum Pregenomic RNA Assay

The pgRNA was detected by RNA simultaneous amplification testing method (HBV-SAT) based on real-time fluorescence detection of isothermal RNA amplification using HBV-SAT kit (Shanghai Rendu Biotechnology Co, Ltd., China) according to the manufacturer’s recommendations. The detailed procedure for serum pgRNA measurement has been described in detail in our previous article (Chen et al., 2019). The linear range was established by testing panels of armored HBV RNA diluted in HBV-negative human serum. The linear concentration ranged from 2 to 8 log copies/mL. The *R*^2^ value of linear equation is more than 0.95. The limit of detection is 50 copies/mL.

### Serum Hepatitis B Core-Related Antigen Assay

The serum HBcrAg level was quantitatively measured using the fully automated CLEIA system (Fujirebio Inc., Tokyo, Japan), and the detailed process of serum HBcrAg measurement is as previously reported (Nassal, 2015). Since the assay’s validated measurement range is from 100 U/mL (2 log10 U/mL) to 10,000,000 U/mL (7 log10 U/mL), serial dilutions of the serum sample are required when serum HBcrAg level is above the detection limit.

### Statistical Analysis

Continuous numerical variables are expressed as the mean ± SD or median (interquartile range, IQR) and categorical variables are expressed as ratios. For categorical variables, the χ^2^ test was used to compare the characteristics of patients with VR and sustained virological response. A *t*-test or nonparametric test was used for continuous variables, as appropriate. Logistic regression analysis was used to assess the association between variables and study endpoints. The predictive accuracy of the risk score model was assessed by the index of concordance and the receiver operating characteristic (ROC) curve over time. *p*-Value less than 0.05 was considered to indicate statistical significance. Statistical analyses were performed using IBM SPSS software version 26.0 and GraphPad Prism 8.0 software.

## Results

### Patient Characteristics

A total of 64 patients were included in this study, with a mean age of 47.33 ± 6.36 years, no patients experienced loss of HBsAg at EOT. All patients were treated for a duration of 60 (42.75–83.25) months with NAs, and 33 (51.6%) patients were treated with first-line agents, including entecavir and tenofovir disoproxil fumarate. Tables 1, 2 show the clinical characteristics of all patients at the start of NAs treatment and at EOT. At the start of NAs treatment, 36 patients (56.3%) were HBeAg positive and 28 (43.7%) were HBeAg negative, HBeAg-positive patients have higher levels of HBV-DNA and HBsAg before the NAs treatment and are treated with NAs for a longer period of time. There was no significant difference in serum HBcrAg levels and pgRNA positivity between the two groups at EOT (Table 1).

**TABLE 1:** Patient characteristics according to baseline HBeAg status.

Factors	HBeAg positive (*N* = 36)	HBeAg negative (*N* = 28)	*P-value*
**Start of treatment**			
Age, years	47.28 ± 6.86	47.39 ± 6.45	0.946
Gender, male/female, *n* (%)	19 (52.8)/17 (47.2)	13 (46.4)/15 (53.6)	0.614
Family history of HBsAg with/not *n* (%)	20 (55.6)/16 (44.4)	16 (57.1)/12 (42.9)	0.889
ALT IU/L	167 (139–216)	190.5 (149–262)	0.180
HBV-DNA log10 IU/mL	7.34 ± 0.14	5.56 ± 0.15	<0.001
HBsAg log10 IU/mL	3.97 ± 0.05	3.71 ± 0.07	0.007
**End of treatment**			
ALT IU/L	22.31 ± 1.46	20.93 ± 1.36	0.505
HBsAg log10 IU/mL	3.41 (2.97–2.47)	3.05 (2.77–3.42)	0.046
HBcrAg log10 U/mL	3.34 ± 0.10	3.08 ± 0.09	0.072
pgRNA positive/negative, *n* (%)	4 (14.3)/32 (85.7)	8 (22.2)/20 (77.8)	0.420
Duration of treatment (month)	72.44 ± 3.85	51.42 ± 2.74	<0.001
**1 year after withdrawal**			
HBeAg changes from negative to positive yes/no *n* (%)	13 (36.1)/23 (63.9)	0/28 (100)	<0.001
VR/non-VR *n* (%)	21 (58.3)/15 (41.7)	12 (42.9)/16 (57.1)	0.219
ALT >40 IU/L	12 (33.3)/24 (66.8)	9 (32.1)/19 (67.9)	0.920

**TABLE 2:** Characteristics comparison between the patients in the VR and non-VR groups.

Factors	All (*n* = 64)	VR (*n* = 33)	Non-VR (*n* = 31)	*P-value*
Age, years	47.33 ± 6.36	47.57 ± 7.52	47.06 ± 5.64	0.759
Gender male/female, *n* (%)	32 (50)/32 (50)	19 (57.6)/14 (42.4)	13 (41.9)/18 (58.1)	0.211
Family history of HBsAg with/not *n* (%)	36 (56.3)/28 (43.7)	18 (54.5)/15 (45.5)	18 (58.1)/13 (41.9)	0.777
**Start of treatment**				
ALT IU/L	188.73 ± 71.48	187.63 ± 70.9	189.90 ± 73.2	0.900
HBV-DNA log10 IU/mL	6.56 ± 1.21	6.68 ± 1.09	6.43 ± 1.33	0.411
HBsAg log10 IU/mL	3.86 ± 0.39	3.87 ± 0.34	3.85 ± 0.43	0.855
HBeAg positive/negative, *n* (%)	36 (56.3)/28 (43.7)	21 (63.6)/12 (36.4)	15 (48.4)/16 (51.6)	0.219
First-line agents/others, *n* (%)	33 (51.6)/31 (48.4)	16 (48.5)/17 (51.5)	17 (54.8)/14 (45.2)	0.611
**End of treatment**				
Duration of treatment (month)	60 (42.75–83.25)	63 (42–85.5)	60 (45–81)	0.397
ALT IU/L	21.70 ± 8.1	21.84 ± 8.14	21.55 ± 8.20	0.884
HBsAg >100/≤100 IU/mL, *n* (%)	57 (89.1)/7 (10.9)	31 (93.9)/2 (6.1)	26 (83.9)/5 (16.1)	0.197
HBcrAg log10 U/mL	3.3 (2.8–3.57)	3.4 (3.3–3.8)	2.8 (2.6–3.2)	<0.001
HBcrAg >3.3/≤3.3 log10 U/mL *n* (%)	33 (51.6)/31 (48.4)	26 (78.8)/7 (21.2)	7 (22.6)/24 (77.4)	<0.001
pgRNA positive/negative *n* (%)	12 (18.8)/52 (81.3)	11 (33.3)/22 (66.7)	1 (3.2)/ 30 (96.8)	0.002
pgRNA positive+ HBcrAg >3.3 log10 U/mL yes/no *n* (%)	9 (14.1)/55 (85.9)	8 (88.9)/25 (45.5)	1 (11.1)/30 (54.5)	0.027
**1 year after withdrawal**				
ALT >40 IU/L	21 (32.8)/43 (67.2)	21 (63.6)/12 (36.4)	0 (0)/31 (100)	<0.001
HBeAg changes from negative to positive yes/no *n* (%)	13 (20.3)/51 (79.7)	13 (39.4)/20 (60.6)	0 (0)/31 (100)	<0.001
HBV-DNA log10 IU/mL		5.85 ± 1.32		

### Comparing Influencing Factors Between the Virological Relapse Patients and Non-virological Relapse Patients

Within 1 year of stopping NAs treatment, 33 patients (51.6%) experienced VR, including 21 (58.3%) patients who were HBeAg positive before the start of treatment and 12 (42.9%) patients who were HBeAg negative before the start of treatment. Of all patients with VR, 21 patients were accompanied by biochemical relapse, the mean HBV-DNA level of the virological relapsed patients was 5.85 ± 1.32 log10 IU/mL. We compared the VR and non-virological relapse (non-VR) patients and found no significant differences between the two groups in terms of age, sex, family history of HBsAg, duration of treatment, and pretreatment ALT. Baseline HBV-DNA level (6.68 ± 1.09 vs. 6.43 ± 1.33 log10 IU/mL, *p* = 0.411) and HBsAg level at the EOT [3.32 log IU/mL (IQR 2.95–3.50) vs. 3.21 log U/mL (IQR 2.80–3.54), *p* = 0.687] had no significant effect on VR. In the VR group, EOT HBcrAg levels were significantly higher than those in the non-VR group [3.4 log U/mL (IQR 3.3–3.8) vs. 2.8 log U/mL (IQR 2.6–3.2), *p* < 0.001]. In addition, EOT pgRNA positive patients were prone to VR (11/12 vs. 22/52, *p* = 0.002). Specific comparisons between the two groups are shown in Table 2.

### Univariate and Multivariable Analysis of Risk Factors for Virological Relapse After Cessation of Nucleos(t)ide Analogs in Patients With Chronic Hepatitis B

**FIGURE 2:**
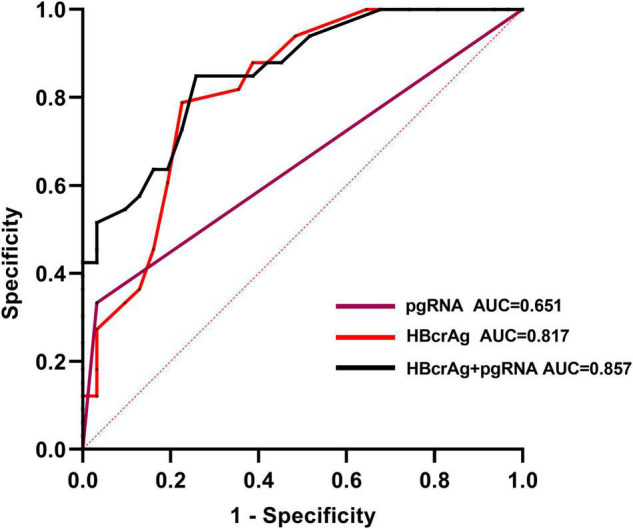
Area under the receiver-operating characteristic curves (AUROC) of EOT pgRNA, EOT HBcrAg and their combination for predicting virological relapse in the cohort. The optimal cut-off value for the EOT HBcrAg for the Youden index to predict virological relapse is 3.3 log10 U/mL (sensitivity of 78% and specificity of 77%).

To identify independent risk factors for VR, univariate and multivariate logistic regression analyses were performed (Table 3). Sex, age, duration of treatment, EOT HBsAg level, and baseline HBV-DNA level and HBeAg status were not associated with VR. Two variables were found to be independent risk factors for VR: EOT pgRNA positivity (OR = 14.59, *p* = 0.026) and higher levels of EOT HBcrAg (OR = 14.14, *p* = 0.001). We used the Youden index to predict the optimal cut-off value of EOT HBcrAg for VR, which measured was 3.25 (3.3) log10 U/mL (sensitivity of 78% and specificity of 77%). Virologic relapse rates were significantly lower in patients with low HBcrAg levels (≤3.3 log10 U/mL) than in those with high levels of HBcrAg (>3.3 log10 U/mL) (7/31 vs. 26/33, *p* < 0.001). Patients with EOT pgRNA positivity and EOT HBcrAg > 3.3 log10 U/mL were more likely to experience VR after discontinuation NAs (88.9 vs. 45.5%, *p* = 0.027). To further assess the value of EOT pgRNA, HBcrAg, and their combination in predicting VR, the area under the receiver operating characteristic curve (AUROC) was calculated for each parameter. The results showed that EOT pgRNA positivity plus EOT higher HBcrAg level had an AUROC value of 0.857 (*p* < 0.001), which was of good predictive value higher than the other parameters (pgRNA positivity, AUC = 0.651, *p* = 0.039. EOT HBcrAg, AUC = 0.817, *p* < 0.001) (Figure 2).

**TABLE 3:** Risk factors for virological relapse after discontinuation of NAs.

Univariate analysis			Multivariable analysis			
Parameter	OR	95% CI	*p*	OR	95% CI	*p*
Age, per year	1.01	(0.93–1.09)	0.750			
Sex (male)	1.88	(0.70–5.07)	0.213			
Family history of HBsAg	0.87	(0.32–2.33)	0.777			
First-line drugs	0.78	(0.29–2.07)	0.611			
**Start of treatment**						
HBeAg positive	1.87	(0.69–5.07)	0.221			
HBV-DNA, per log10 IU/mL	1.19	(0.79–1.80)	0.405			
HBsAg, per log10 IU/mL	1.13	(0.32–4.03)	0.852			
**End of treatment**						
Duration of treatment (month)	1.01	(0.96–1.03)	0.515			
HBcrAg per log10 U/mL	15.91	(3.76–70.72)	<0.001	14.14	(3.08–65.03)	0.001
HBcrAg >3.3 log10 U/mL	12.73	(3.89–41.67)	<0.001			
PgRNA positive	15.00	(1.80–124.93)	0.012	14.59	(1.38–153.89)	0.026

### Characteristics of Patients Whose Serum Hepatitis B e Antigen Changes From Negative to Positive After Discontinuation of Nucleos(t)ide Analogs

**FIGURE 3:**
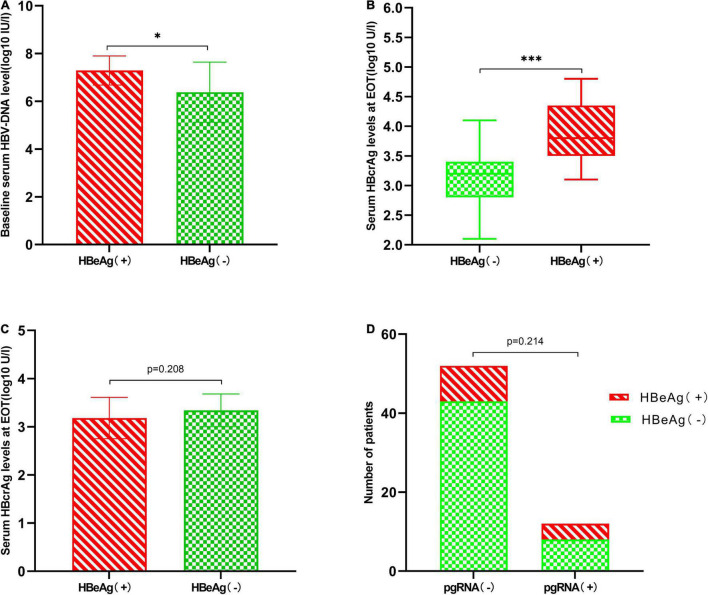
Characteristics of patients whose serum HBeAg changes from negative to positive after 1 year of discontinuation of NAs. **(A)** Patients who experienced HBeAg changes from negative to positive have higher baseline HBV-DNA levels (7.29 ± 0.61 vs. 6.38 ± 1.26 log10 IU/mL, *p* = 0.01). **(B)** Patients who experienced HBeAg changes from negative to positive had higher EOT HBcrAg levels [3.80 log U/mL (IQR 3.50–4.35) vs. 3.20 log U/mL (IQR 2.80–3.40), *p* < 0.001]. **(C)** Comparison of EOT HBsAg levels between two groups (3.18 ± 0.43 vs. 3.34 ± 0.34 log10 IU/mL, *p* = 0.208). **(D)** EOT pgRNA status has no significant effect on HBeAg conversion (4/12 vs. 9/52, *p* = 0.214). *P < 0.05 and ***P < 0.001.

A total of 13 (20.3%) patients experienced a conversion in serum HBeAg from negative at EOT to positive within 1 year of discontinuation of NAs, and those patients were HBeAg positive before the start of treatment. We analyzed the characteristics of these patients and found that baseline HBV-DNA levels (7.29 ± 0.61 vs. 6.38 ± 1.26 log10 IU/mL, *p* = 0.01), EOT HBcrAg levels (3.88 ± 0.49 vs. 3.06 ± 0.46 log10 U/mL, *p* < 0.001) (Figures 3A,B) were significantly higher than patients with continued HBeAg negative status. But we found no significant differences in age, gender, family history of HBsAg, duration of treatment, EOT HBsAg levels, and EOT pgRNA status between the two groups (Table 4 and Figures 3C,D).

**TABLE 4:** Characteristics of patients whose serum HBeAg changes from negative to positive after 1 year of discontinuation of NAs.

Factors	HBeAg converted to positive (*N* = 13)	HBeAg persistently negative (*N* = 51)	*P-Value*
Age, years	49.69 ± 8.71	46.73 ± 5.95	0.120
Gender, male/female, *n* (%)	9 (69.2)/4 (30.8)	23 (45.1)/28 (54.9)	0.614
Family history of HBsAg with/not *n* (%)	4 (30.8)/9 (69.2)	24 (47.1)/27 (52.9)	0.291
**Start of treatment**			
HBeAg positive/negative *n* (%)	13 (100)/0	28 (54.9)/23 (45.1)	<0.001
HBV-DNA log10 IU/mL	7.29 ± 0.61	6.38 ± 1.26	0.01
HBsAg log10 IU/mL	3.97 (3.17–4.97)	3.85 (3.55–3.98)	0.161
**End of treatment**			
HBsAg log10 IU/mL	3.18 ± 0.43	3.34 ± 0.34	0.208
HBcrAg log10 U/mL	3.88 ± 0.49	3.06 ± 0.46	<0.001
pgRNA positive/negative, *n* (%)	4 (30.8)/9 (69.2)	8 (15.7)/43 (84.3)	0.214
Duration of treatment (month)	70.92 ± 22.61	61.29 ± 22.04	0.167

## Discussion

Discontinuation of NAs for HBV patients remains a difficult clinical issue. Although NAs significantly inhibit HBV replication and reduce serum HBV DNA to undetectable levels, complete elimination of HBV virus from hepatocytes is difficult due to the persistence of cccDNA in the nucleus of infected cells (Wang et al., 2016). After discontinuation of NAs, cccDNA has the risk of reactivation, resulting in a higher rate of VR, with a virological recurrence rate of 51.6% 1 year in our study. Dynamic monitoring of intrahepatic cccDNA levels can predict sustained virological response after cessation of NAs therapy. However, the invasive nature of dynamic liver biopsy and the potential for sampling error have greatly limited the use of intrahepatic cccDNA in actual clinical practice. Therefore, we need to identify the key factors of VR. Influencing factors such as age, sex, baseline HBV DNA, EOT HBsAg, and duration of treatment have been demonstrated to be associated with VR in previous studies (Chen et al., 2015; Jung et al., 2016; Hsu et al., 2019). However, our study showed no significant relationship between these factors and VR, suggesting that there are discrepancies between studies. We need more accurate and consistent indicators to guide safe drug discontinuation, serum HBcrAg and PgRNA are novel virological indicators, this prospective study demonstrates the significant role of two novel indicators in patients with chronic HBV infection, especially in predicting VR after drug discontinuation, with an AUROC value of 0.857 (*p* < 0.001). which is in line with most studies (Jaroenlapnopparat et al., 2021).

HBV-DNA is the most commonly used virological marker in clinical practice and has long been widely used to assess the activity of viral replication and the efficacy of NAs in treating patients with CHB. However, NAs act on only a limited number of steps in the viral replication cycle and do not affect cccDNA. Therefore, HBV-DNA does not reflect the true status of cccDNA in hepatocytes in the presence of long-term antiviral drugs. A Korean study showed that HBV DNA >2,000,000 IU/mL before starting treatment in HBeAg-positive patients was an independent risk factor for VR (Jung et al., 2016) (OR = 9.285, *p* = 0.036). A Taiwan study also demonstrated that baseline HBV-DNA was a risk factor for VR in patients with HBsAg below 100 IU/mL (Tseng et al., 2020). However, in line with some studies (Tsuge et al., 2013), no significant effect of baseline HBV-DNA on VR was shown in our study.

Serum HBsAg levels correlate with hepatic cccDNA activity and the loss of serum HBsAg is an ideal indicator to evaluate safety discontinuation. Previous studies have shown that EOT serum HBsAg levels are an independent risk factor for VR, but predominantly at low levels, with EOT HBsAg >100 or >40 IU/L (Hsu et al., 2019; Fan et al., 2020; Tseng et al., 2020; Xie et al., 2021). In our study, although EOT HBsAg was not significantly associated with VR, patients with EOT HBsAg ≤100 IU/mL had a lower rate of VR than those with EOT HBsAg >100 IU/mL [28.6% (2/7) vs. 54.4% (31/57), *p* = 0.197], which is consistent with the results of previous studies. This result may be due to the low number of patients with low EOT HBsAg levels in our cohort.

Intrahepatic pgRNA is transcribed from cccDNA and correlates strongly with intrahepatic cccDNA activity, but detection of intrahepatic pgRNA is difficult and relies on liver puncture biopsy. Therefore, serum HBV RNA is a promising marker of cccDNA transcriptional activity. Especially during NAs treatment, serum HBV DNA levels are often undetectable and serum pgRNA is a better reflection of the status of intrahepatocellular cccDNA (Giersch et al., 2017). It has been shown that serum pgRNA is an independent predictor of viral rebound after discontinuation of treatment (Tsuge et al., 2013; Wang et al., 2016). A prospective study from China showed that 6 years after stopping treatment with NA, all patients with EOT pgRNA levels ≥20,000 copies/mL experienced biochemical relapse, compared to 23.8% of patients with HBV RNA levels <1,000 copies/mL (*p* < 0.001) (Xia et al., 2021). In our study it was shown that patients who were negative for EOT pgRNA were more likely to have a sustained virological response, while those who were EOT pgRNA positive were more likely to have a VR (11/12 vs. 22/54, *p* = 0.002). This result was consistent with previous studies. In addition, EOT pgRNA positivity was found to be an independent risk factor for VR in a multivariate regression analysis (OR = 14.59, *p* = 0.026) with an AUROC value of 0.651.

Hepatitis B core-related antigen is a novel serum complex viral protein that consists of three related proteins sharing the same 149 amino acid sequence, including HBcrAg, HBeAg and a truncated 22 kDa precore protein (p22Cr) (Mak et al., 2018). In our previous study, it was demonstrated that serum HBcrAg is a better indicator of cccDNA than pgRNA and HBsAg (Chen et al., 2019). Studies have also reported that HBcrAg can be detected even when serum HBV DNA becomes undetectable or HBsAg is lost (Seto et al., 2014). In a Taiwanese study, the independent risk factor of VR was EOT HBcrAg >4 log U/mL (Huang et al., 2021). Jung et al. (2016) showed that EOT HBcrAg >3.7 log U/mL was independently associated with more than three-times higher risk of VR in HBeAg-negative CHB patients. In our study, HBcrAg levels in the VR group were higher than those in N-VR group. In multivariate regression analysis, EOT higher HBcrAg level was an independent risk factor, and optimal cut-off value of EOT HBcrAg for VR is 3.3 log10 U/mL, Patients with EOT HBcrAg >3.3 log10 U/mL are more likely to experience VR. This suggests that the lower the EOT HBcrAg level, the lower the risk of VR after drug discontinuation.

Our study has a number of limitations. Firstly, the sample size was relatively small and the follow-up period was short, limited to 1 year. Secondly, this is a single-center study and multiple studies are needed to confirm our main findings. Thirdly, our study only included Asian populations. Fourth, the small sample size did not allow for separate analysis of E antigen-positive and E antigen-negative patients.

## Conclusion

According to current discontinuation criteria, higher relapse rates occur after cessation of NAs. EOT HBcrAg and PgRNA levels have the potential to predict VR in patients with chronic HBV infection who discontinue NAs therapy. Low HBcrAg levels at the EOT and negative PgRNA are associated with a lower risk of relapse. Combining these two novel indicators could guide CHB patients in choosing the appropriate time to stop NAs.

## Data Availability Statement

The raw data supporting the conclusions of this article will be made available by the authors, without undue reservation.

## Ethics Statement

The studies involving human participants were reviewed and approved by the West China Hospital Ethics Committee. The patients/participants provided their written informed consent to participate in this study.

## Author Contributions

E-QC contributed to the study concept and design. F-DW, JZ, L-QL, M-LW, Y-CT, Y-HW, and D-MZ contributed to the data acquisition. F-DW contributed to the data analysis and manuscript drafting. All authors approved the final version of the manuscript.

## Conflict of Interest

The authors declare that the research was conducted in the absence of any commercial or financial relationships that could be construed as a potential conflict of interest.

## Publisher’s Note

All claims expressed in this article are solely those of the authors and do not necessarily represent those of their affiliated organizations, or those of the publisher, the editors and the reviewers. Any product that may be evaluated in this article, or claim that may be made by its manufacturer, is not guaranteed or endorsed by the publisher.
